# Anti-Inflammatory Phenolic Metabolites from the Edible Fungus *Phellinus baumii* in LPS-Stimulated RAW264.7 Cells

**DOI:** 10.3390/molecules22101583

**Published:** 2017-09-21

**Authors:** Seulah Lee, Dahae Lee, Tae Su Jang, Ki Sung Kang, Joo-Won Nam, Hae-Jeung Lee, Ki Hyun Kim

**Affiliations:** 1 School of Pharmacy, Sungkyunkwan University, Suwon 16419, Korea; sarahlee0801@gmail.com (S.L.); pjsldh@naver.com (D.L.); 2 College of Korean Medicine, Gachon University, Seongnam 13120, Korea; kkang@gachon.ac.kr; 3 Institute of Green Bio Science & Technology, Seoul National University, Pyeong Chang 232-916, Korea; jangts@snu.ac.kr; 4 College of Pharmacy, Yeungnam University, Gyeongsan, Gyeongbuk 38541, Korea; jwnam@yu.ac.kr; 5 Department of Food and Nutrition, Gachon University, Seongnam 13120, Korea; skysea@gachon.ac.kr

**Keywords:** *Phellinus baumii*, Hymenochaetaceae, bioactivity-guided isolation, anti-inflammation, NO, NF-kappaB

## Abstract

The edible fungus *Phellinus baumii* Pilat (Hymenochaetaceae) has been used in Korean traditional medicines for strengthening health and prolonging life. An extract of the fruiting bodies of *P. baumii* was subjected to bioassay-guided fractionation based on its anti-inflammatory effects in lipopolysaccharide (LPS)-stimulated RAW264.7 cells. The resulting fractions were chemically investigated, leading to isolation of three phenolic compounds (**1**–**3**), a sesquiterpene (**4**), two steroids (**5**–**6**), a fatty acid (**7**), and a cerebroside (**8**). Spectroscopic analyses including 1D and 2D NMR spectroscopy and LC/MS were used to determine their chemical structures. Compounds **2**, **4**, **5**, **7** and **8** were identified in *P. baumii* for the first time. Since all compounds were isolated from active fractions with anti-inflammatory activity, their ability to inhibit LPS-stimulated nitric oxide (NO) production in RAW264.7 cells were evaluated in vitro. Compounds **1**, **2**, **3**, **5** and **7** inhibited LPS-stimulated NO production, and compounds **1**–**3** had IC_50_ values <10 μM. Treatment of LPS-stimulated RAW264.7 cells with compounds **1**–**3** inhibited phosphorylation of IKKα and IκBα. In addition, treatment of compounds **1**–**3** reduced LPS-induced increases of nuclear factor-kappa B (NF-κB) p65, iNOS and COX-2 protein expressions. Collectively, compounds **1**–**3** inhibited NF-κB-dependent inflammation in RAW264.7 cells. Thus, *P. baumii* is a potential source of natural anti-inflammatory agents, and active compounds **1**–**3** could be promising lead compounds for the development of novel anti-inflammatory agents.

## 1. Introduction

*Phellinus baumii* Pilat, a fungus belonging to the family Hymenochaetaceae, is an edible mushroom commonly known as “Sanghuang” in Korea [1,2]. The fruiting bodies of *P. baumii* have traditionally been included in the diet and in medicines in Asian countries such as Korea, China, and Japan [1,2,3,4,5]. *P. baumii* and other mushrooms of the genus *Phellinus* are well known to strengthen health and prolong life [6,7,8,9,10]. The traditional use of *P. baumii* has been verified both in vivo and in vitro [11,12,13], which provides strong evidence supporting its expanded use in the development of functional foods.

Extracts of *P. baumii* have been identified as potential modulators of oxidative stress, inflammation, and immunity, and have been reported to have various biological activities, including anti-obesity, anti-platelet, and hypoglycemic properties [7,10,12,13,14,15]. Although many chemical analyses have been published on *Phellinus* fungi such as *P. linteus*, the chemical constituents and significant metabolites of *P. baumii* have been relatively uninvestigated. Previous studies have demonstrated that polysaccharides isolated from *P. baumii* can inhibit tumor growth and metastasis [7,15], and other reports have noted the nuclear factor-kappa B (NF-κB) inhibitory effects of phenolic compounds and anti-influenza activities of polyphenols isolated from *P. baumii* [10,16].

In our continuing efforts to discover bioactive secondary metabolites from Korean wild mushrooms [17,18,19,20,21,22], we have found that an ethanolic (EtOH) extract of the fruiting bodies of *P. baumii* can inhibit nitric oxide (NO) production in lipopolysaccharide (LPS)-stimulated RAW264.7 cells. In this study, we used a bioactivity-guided isolation technique to identify metabolites from this EtOH extract with anti-inflammatory activity in LPS-stimulated RAW264.7 cells. Our chemical analysis led to the isolation and identification of three phenolic compounds (**1**–**3**), a sesquiterpene (**4**), two steroids (**5**–**6**), a fatty acid (**7**), and a cerebroside (**8**) with anti-inflammatory activity from the active fractions. Herein, we report the bioactivity-guided isolation and structural elucidation of compounds **1**–**8**, along with their anti-inflammatory activities and underlying mechanisms of action.

## 2. Results & Discussion

### 2.1. Bioactivity-Guided Fractionation for Anti-Inflammatory Effects

**Figure 1:**
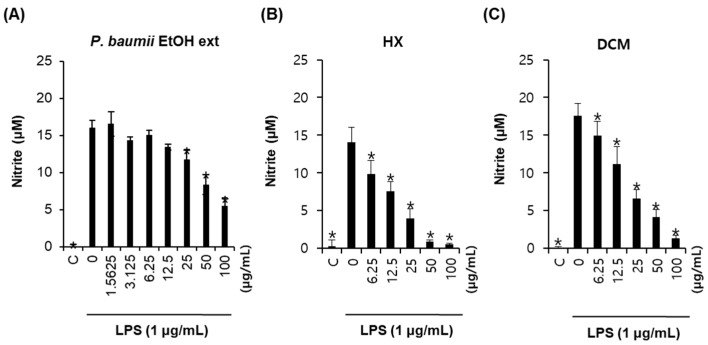
Anti-inflammatory effects of the EtOH extract of *P. baumii* and its fractions (hexane (HX) and dichloromethane (DCM) fractions): inhibition of lipopolysaccharide (LPS)-induced NO production in RAW264.7 mouse macrophages. (**A**–**C**) Inhibitory effects of the EtOH extract of *P. baumii* (**A**) and its hexane (**B**) and dichloromethane (**C**) fractions on LPS-induced NO production in RAW264.7 mouse macrophages. The cells were pretreated with the indicated concentrations of samples for 2 h and then exposed to 1 μg/mL LPS for 24 h. * *p* < 0.05 compared to the LPS-treated value.

Dried and chopped fungal material was extracted with 60% aqueous EtOH at room temperature and then filtered. The filtrate was evaporated under reduced pressure with a rotavapor to obtain a crude EtOH extract. In our screening test, the EtOH extract inhibited NO production in a dose-dependent manner in LPS-stimulated RAW264.7 cells, with an IC_50_ value of 56.9 ± 1.2 μg/mL, and no significant cell death was observed up to the concentration of 100 μg/mL (Figure 1).

The EtOH extract was then solvent-partitioned for bioassay-guided fractionation with hexane, dichloromethane (CH_2_Cl_2_), ethyl acetate (EtOAc), and *n*-butanol, yielding soluble fractions of hexane (HX), CH_2_Cl_2_ (DCM), EtOAc (EA), and *n*-butanol (BuOH), respectively. Prior to investigating the anti-inflammatory effects of the four solvent-partitioned fractions, we examined the cellular toxicity of these fractions in RAW264.7 cells with MTT assay [23,24,25,26]. No significant cell death was observed for the HX, DCM, and BuOH fractions up to concentrations of 50 μg/mL, while the EA fraction caused cell death at a concentration of 50 μg/mL (data not shown). The effects of the three fractions (HX, DCM, and BuOH fractions) without cellular toxicity on NO level were assessed in LPS-stimulated RAW264.7 cells (Figure 1). The HX and DCM fractions significantly inhibited NO production in a dose-dependent manner, with IC_50_ values of 13.8 ± 1.2 μg/mL and 18.9 ± 1.7 μg/mL, respectively, while the BuOH fraction had no effect. These results inspired us to thoroughly investigate the two active fractions for anti-inflammatory constituents that inhibited NO production.

### 2.2. Chemical Investigation and Identification of the Active Compounds

**Figure 2:**
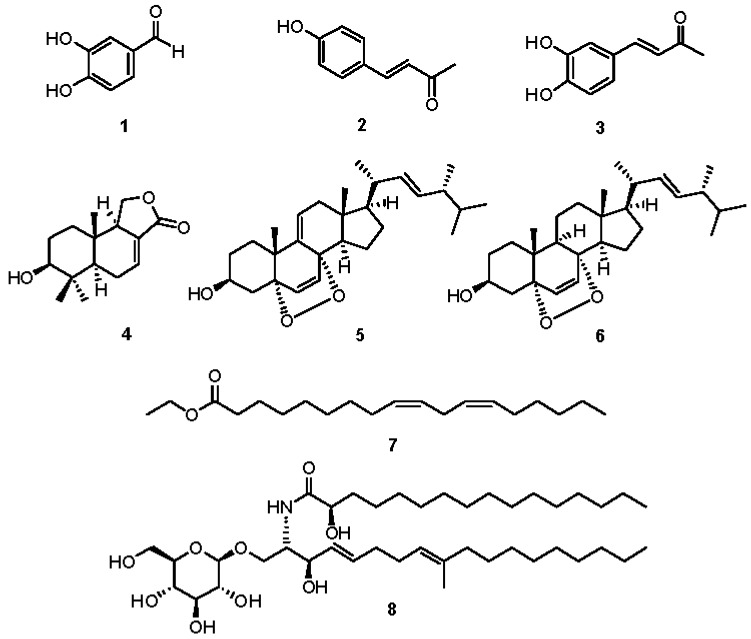
Chemical structures of compounds **1**–**8**.

The DCM fraction was subjected to repeated column chromatography and semi-preparative HPLC purification, which resulted in the isolation of five compounds (**1**–**4** and **8**). The HX fraction was chemically analyzed using the same method, which led to the isolation of three compounds (**5**–**7**). The structures of the isolates were determined using spectroscopic methods including 1D and 2D NMR spectroscopy and LC/MS (Figure 2). Compounds **1**–**8** were identified as 3,4-dihydroxybenzaldehyde (**1**) [27], 4-(4-hydroxyphenyl)-3-buten-2-one (**2**) [28], 4-(3,4-dihydroxyphenyl)-3-buten-2-one (**3**) [27], 3β-hydroxycinnamolide (**4**) [29], 9,11-dehydroergosterol peroxide (**5**) [30], ergosterol peroxide (**6**) [30], ethyl linoleate (**7**) [31], and (2*R*)-*N*-[(1*S*,2*R*,3*E*,7*E*)-1-[(β-d-glucopyranosyloxy)methyl]-2-hydroxy-8-methyl-3,7-heptadecadien-1-yl]-2-hydroxy-hexadecanamide (**8**) [32] through comparison of their obtained spectroscopic data with reported values, as well as LC/MS analysis. To the best of our knowledge, compounds **2**, **4**, **5**, **7** and **8** were identified from *P. baumii* for the first time in this study.

### 2.3. Effects of Compounds ***1***–***8*** on NO Production

**Figure 3:**
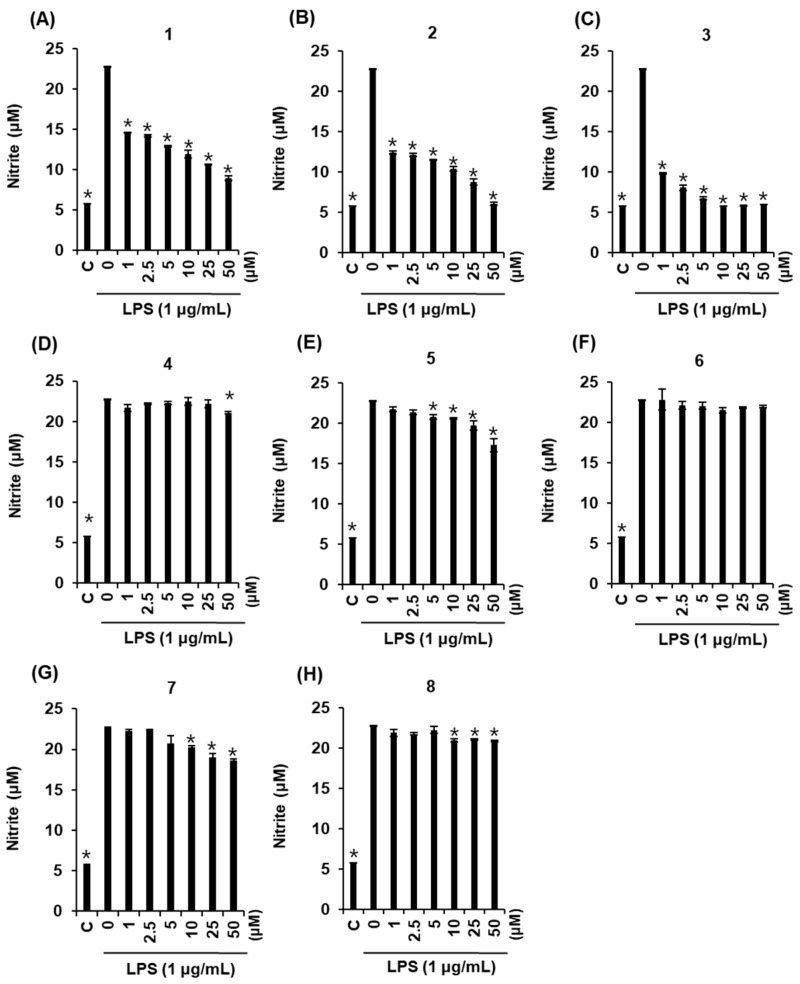
Anti-inflammatory effects of the isolated compounds: inhibition of LPS-induced NO production in RAW264.7 mouse macrophages. Inhibitory effects of isolated compounds **1** (**A**), **2** (**B**), **3** (**C**), **4** (**D**), **5** (**E**), **6** (**F**), **7** (**G**), and **8** (**H**) on LPS-induced NO production in RAW264.7 mouse macrophages. The cells were pretreated with the indicated concentrations of samples for 2 h and then exposed to 1 μg/mL LPS for 24 h. * *p* < 0.05 compared to the LPS-treated value.

Since all isolated compounds (**1**–**8**) were purified from active fractions that inhibited NO production, these compounds were then tested individually for their ability to inhibit NO production in LPS-activated RAW264.7 macrophages as a measure of their anti-inflammatory effects. No cytotoxic effects were noted for compounds **1**–**8** at concentrations up to 50 μM (data not shown), and these non-toxic concentrations were used for subsequent anti-inflammatory activity tests. Compounds **1**, **2**, **3**, **5** and **7** inhibited LPS-stimulated NO production (Figure 3). Among them, compounds **1**, **2** and **3** significantly inhibited NO production in LPS-activated RAW264.7 macrophages, with IC_50_ values of 9.1 ± 0.1, 0.8 ± 0.1, and 0.7 ± 0.2 μM, respectively. Further mechanistic studies were performed with these three compounds, since their IC_50_ values were less than 10 μM.

### 2.4. Effects of Compounds ***1***–***3*** on Proteins Associated with Inhibition of NF-κB in LPS-Stimulated RAW264.7 Mouse Macrophages

**Figure 4:**
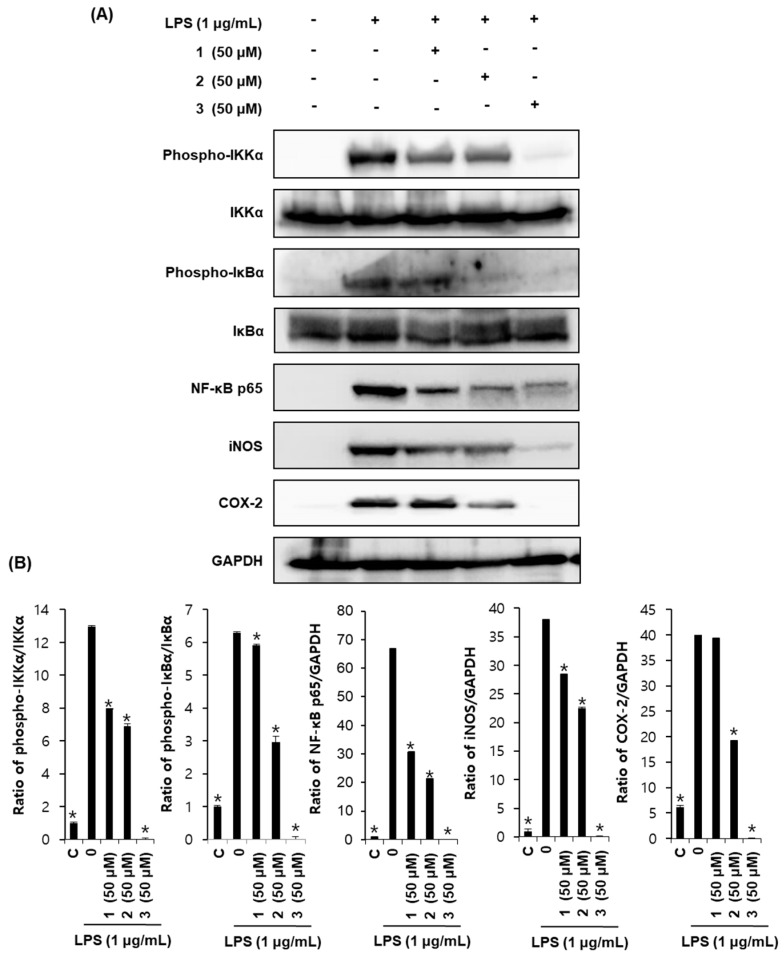
Effects of compounds **1**, **2** and **3** on the expression of proteins associated with inhibition of NF-κB in LPS-stimulated RAW264.7 mouse macrophages (**A**). Each bar graph presents the densitometric quantification of western blot bands (**B**). * *p* < 0.05 compared to the LPS-treated value.

To further confirm the anti-inflammatory properties of compounds **1**, **2** and **3**, we investigated their effects on the phosphorylation of IKKα and IκBα, and protein expressions of NF-κB p65, iNOS, and COX-2, which are involved in the pathogenesis of chronic inflammatory diseases. In un-activated cells, NF-κB localizes to the cytoplasm, and the IκB proteins bind to NF-κB and inhibit its nuclear localization and activation signals [33,34]. The phosphorylation of ΙκB proteins is a key step in the activation of NF-κB and is regulated by IκB kinases (IKKs) [35], while IKK activity is induced by activators of the NF-κB pathways [33,34]. Compounds **1**, **2** and **3** inhibited the phosphorylation of IKKα and IκBα in LPS-stimulated RAW264.7 cells. Collectively, compounds **1**, **2**, and **3** inhibited the NF-κB-dependent inflammation pathway in RAW264.7 cells (Figure 4).

NF-κB also activates the expression of enzymes that contribute to the pathogenesis of the inflammatory process, including iNOS and COX-2, the latter of which generates prostanoids [36]. Compounds **1**, **2** and **3** also significantly reduced the expression of iNOS and COX-2 (Figure 4), reflecting the inhibition of the expression of these inflammatory enzymes by NF-κB deactivation.

The anti-inflammatory components of *P. baumii* have not been widely studied yet. Recently, a related study described the phenolic compounds of *P. baumii* with NF-κB inhibitory effects in PC-3 cells [10]. These phenolic compounds (dihydroflavones and hispidin derivatives) were examined for their inhibitory effects on NF-κB activity in a luciferase gene reporter assay, and the IC_50_ values of the active compounds were approximately 20 μM higher than those of active phenolic compounds identified in the present study [10]. Ultimately, we identified components of *P. baumii* with higher effectiveness in anti-inflammation.

## 3. Materials and Methods

### 3.1. General Experimental Procedures

Ultraviolet (UV) spectra were acquired on an Agilent 8453 UV-visible spectrophotometer (Agilent Technologies, Santa Clara, CA, USA). NMR spectra were obtained with a Bruker AVANCE III 700 NMR spectrometer operating at 700 MHz (^1^H) and 175 MHz (^13^C) (Bruker, Billerica, MA, USA). Semipreparative HPLC was conducted with a Shimadzu Prominence HPLC System equipped with SPD-20A/20AV-series Prominence HPLC UV-Vis detectors (Shimadzu, Tokyo, Japan). LC/MS analysis was performed on an Agilent 1200-series HPLC system with a diode array detector and a 6130-series ESI mass spectrometer with an analytical Kinetex C18 100 Å column (100 mm × 2.1 mm i.d., 5 μm) (Phenomenex, Torrance, CA, USA). Column chromatography was performed with silica gel 60, 230–400 mesh (Merck, Darmstadt, Germany). Thin-layer chromatography (TLC) was conducted with precoated silica gel F_254_ plates and reverse-phase (RP)-18 F_254s_ plates (Merck, Kenilworth, NJ, USA). Spots on TLC were detected with UV light and heat after the plates were dipped in anisaldehyde-sulfuric acid.

### 3.2. Fungus Material

Fresh fruiting bodies of *P. baumii* were purchased from DDLE A CHE Co., Ltd. (Cheonan-si, Chungcheongnam-do, Korea), having been cultivated in Jungdo-ri, Sanyang-myeon, Munkyung, Gyeongsangbuk-do, Korea, in April 2016. Samples of fungal material were identified by one of the authors (Hae-Jeung Lee). A voucher specimen (SKKU 2016-04-SH) has been deposited in the herbarium of the School of Pharmacy, Sungkyunkwan University, Suwon, Korea.

### 3.3. Extraction, Fractionation, and Purification Methods

The dried and chopped fungal material (150 g) was extracted with 60% aqueous EtOH three times (each 3 L × 24 h) at room temperature. Extracts were filtered, and the filtrate was evaporated under reduced pressure with a rotavapor to obtain a crude EtOH extract (10.0 g). The extract was suspended in distilled water (700 mL) and MeOH (30 mL) and successively solvent-partitioned with hexane, dichloromethane, ethyl acetate, and *n*-butanol, yielding soluble layers of hexane (HX) (0.9 g), CH_2_Cl_2_ (DCM) (0.5 g), EtOAc (EA) (0.4 g), and *n*-butanol (BuOH) (1.1 g). The HX and DCM fractions significantly inhibited NO production in a dose-dependent manner in LPS-stimulated RAW264.7 cells.

The DCM fraction (0.5 g) was subjected to silica gel column chromatography (CC) (CH_2_Cl_2_/MeOH, from 100:1 to 1:1), yielding 6 fractions (Fr. A1–A6). Fr. A4 (23.5 mg) was purified by semi-preparative HPLC (32% MeOH) with a Phenomenex Luna phenyl-hexyl column (250 mm × 10 mm i.d., flow rate: 2 mL/min) to yield compounds **1** (*t_R_* 18.0 min, 2.8 mg) and **3** (*t_R_* 37.5 min, 3.3 mg). Fr. A2 (22.7 mg) was also purified by semi-preparative HPLC (gradient solvent system from 50% MeOH to 100% MeOH) with a Phenomenex Luna phenyl-hexyl column (250 mm × 10 mm i.d., flow rate: 2 mL/min) to yield compounds **2** (*t_R_* 17.0 min, 1.1 mg) and **4** (*t_R_* 24.0 min, 1.7 mg). Compound **8** (*t_R_* 39.0 min, 0.7 mg) was purified from Fr. A5 (41.5 mg) using the same method.

The HX fraction (0.9 g) was subjected to silica gel CC (hexane/EtOAc, from 5:1 to 1:1) to yield 5 fractions (Fr. B1–B5). Fr. B4 (14.2 mg) was purified by semi-preparative HPLC (92% MeOH) with a Phenomenex Luna phenyl-hexyl column (250 mm × 10 mm i.d., flow rate: 2 mL/min), and compounds **5** (*t_R_* 29.0 min, 0.6 mg) and **6** (*t_R_* 34.5 min, 1.6 mg) were obtained. Fr. B1 (15.8 mg) was separated on a Phenomenex Luna phenyl-hexyl column (250 mm × 10 mm i.d., flow rate: 2 mL/min) in a semi-preparative HPLC system (gradient solvent system from 80% MeOH to 100% MeOH) to yield compound **7** (*t_R_* 43.0 min, 2.6 mg).

### 3.4. Cell Culture and MTT Cell Viability Assay

RAW264.7 mouse macrophages were purchased from the American Type Culture Collection (Rockville, MD, USA). The cells were cultured in DMEM (Cellgro, Manassas, VA, USA) supplemented with 10% FBS, 1% penicillin/streptomycin (Invitrogen Co., Grand Island, NY, USA), and 4 mM l-glutamine in an atmosphere of 5% CO_2_ at 37 °C. Cell viability was determined with an Ez-Cytox cell viability detection kit. When the cells were approximately 80% confluent, they were seeded in 96-well culture plates at 1 × 10^5^ cells per well and incubated for 24 h for adhesion. Then cells were treated with the control (0.5% DMSO) or with indicated concentrations of the EtOH extract of *P. baumii*, its fractions, and isolated compounds **1**–**8**. After cells had been incubated with these treatments for 24 h, 10 μL of Ez-Cytox reagent was added to each well and incubated for 2 h. Cell viability was determined from the absorbance at 450 nm measured with a microplate reader (PowerWave XS; Bio-Tek Instruments, Winooski, VT, USA).

### 3.5. Measurement of Nitric Oxide Production

*Escherichia coli* LPS (strain 055:B5) was purchased from Sigma Aldrich (Saint Louis, MO, USA). NO production was determined by the Griess reaction, which measures the accumulation of nitrite in the culture medium. When the cells were approximately 80% confluent, they were seeded in 96-well culture plates at 1 × 10^5^ cells per well and incubated for 24 h for adhesion. The cells were then pretreated with phenol red-free medium containing the control (0.5% DMSO) or the indicated concentrations of compounds **1**–**8** for 2 h and then exposed to 1 μg/mL *E. coli* LPS (strain 055:B5) for 24 h. The supernatant (80 μL) was collected, mixed with an equal volume (80 μL) of Griess reagent (1% sulfanilamide, 5% phosphoric acid, and 0.1% *N*-(1-naphthyl)-ethylenediamine), and incubated in the dark at room temperature for 10 min. The absorbance at 540 nm was measured with a microplate reader (PowerWave XS; Bio-Tek Instruments, Winooski, VT, USA). Sodium nitrite was used to generate a standard reference curve.

### 3.6. Western Blotting Analysis

RAW264.7 cells seeded in 6-well plates were treated with compounds **1**, **2** and **3** (50 μM). After the cells were incubated with these treatments for 2 h, 1 μg/mL *E. coli* LPS (strain 055:B5) was added to each well and incubated for 24 h. Then cells were lysed with RIPA buffer (20 mM Tris-HCl (pH 7.5), 150 mM NaCl, 1 mM Na_2_EDTA, 1 mM EGTA, 1% NP-40, 1% sodium deoxycholate, 2.5 mM sodium pyrophosphate, 1 mM beta-glycerophosphate, 1 mM Na_3_VO_4_, 1 µg/mL leupeptin, Cell Signaling, MA, USA) supplemented with 1 mM phenylmethylsulfonyl fluoride (PMSF) immediately before use. Protein concentrations were determined with a Pierce™ BCA Protein Assay Kit (Thermo Scientific, Waltham, MA, USA). Equal amounts (20 μg/lane) of protein (whole-cell extracts) were separated by electrophoresis in a 10% sodium dodecyl sulfate-polyacrylamide gel and transferred onto PVDF transfer membranes. Specific proteins were analyzed with epitope-specific primary antibodies to NF-κB p65 (#4764), iNOS (#13120), COX-2 (#4842), IκBα (#9242), phospho-IkBα (#2859), IKKα (#2682), phospho-IKKα (#2697), glyceraldehyde 3-phosphate dehydrogenase (#2118), and horseradish peroxidase (HRP)-conjugated anti-rabbit antibodies (Cell Signaling, Boston, MA, USA). Bound antibodies were detected with ECL Advance Western Blotting Detection Reagents (GE Healthcare, Little Chalfont, UK) and visualized with a FUSION Solo Chemiluminescence System (PEQLAB Biotechnologie GmbH, Erlangen Germany).

### 3.7. Statistical Analysis

Statistical significance was determined using analysis of variance followed by multiple comparison tests with Bonferroni adjustment. A *p* value of less than 0.05 was considered statistically significant.

## 4. Conclusions

In the current study, we found that an EtOH extract of the fruiting bodies of *P. baumii* inhibited NO production in LPS-activated RAW264.7 macrophages. This EtOH extract was thus examined for its active constituents, and eight compounds were identified (**1**–**8**), five of which (**2**, **4**, **5**, **7** and **8**) were identified in the fungus *P. baumii* for the first time. Among the compounds identified in this work, compounds **1**, **2** and **3** significantly inhibited NO production in LPS-activated RAW264.7 macrophages, with IC_50_ values of 9.1 ± 0.1, 0.8 ± 0.1 and 0.7 ± 0.2 μM, respectively. These three active compounds inhibited the phosphorylation of IKKα and IκBα. In addition, treatment of compounds **1**–**3** reduced LPS-induced increases of NF-κB p65, iNOS, and COX-2 protein expressions. These findings provide experimental evidence that compounds **1**, **2** and **3** could contribute to the health benefits of *P. baumii* as an anti-inflammatory agent.
